# Redesign of Translocon EXP2 Nanopore for Detecting Peptide Fragments

**DOI:** 10.1002/smtd.202401562

**Published:** 2025-02-05

**Authors:** Mitsuki Miyagi, Misa Yamaji, Nina Kurokawa, Masafumi Yohda, Ryuji Kawano

**Affiliations:** ^1^ Department of Biotechnology and Life Science Tokyo University of Agriculture and Technology Tokyo 184‐8588 Japan

**Keywords:** lipid bilayer, microfluidics, nanopore, peptide sensing, translocon

## Abstract

Nanopore sensing is a rapid, label‐free technique that enables single‐molecule detection and is successfully applied to nucleic acid sequencing. Extending this technology to the detection and sequencing of peptides and proteins is a key area of interest. However, the complex structures and diverse charge distributions of peptides and proteins present challenges for extensive detection using existing nanopores. In this study, the focus is on the EXP2 nanopore derived from the malaria parasite *Plasmodium falciparum* to address these challenges. Previously, it is characterized wild‐type EXP2 (WT‐EXP2) nanopores and demonstrated their ability to detect polypeptides, although intrinsic electrical noise from the pore posed difficulties for accurate detection. To overcome these limitations, several EXP2 nanopore mutants are designed, including EXP2_ΔD231_, EXP2_NC_, and EXP2_NC_^K42D/S46F^, to reduce electrical noise and improve peptide detection accuracy. The EXP2_ΔD231_ mutant reduced electrical noise by more than 50% compared to WT‐EXP2 and improved the discrimination accuracy of oligoarginine peptides. In addition, the EXP2_ΔD231_ detected and discriminated eight different peptides, ranging in molecular weight from small to large, that are previously challenging to detect using a single nanopore type. These results suggest that engineered EXP2 nanopores could serve as effective tools for peptide and protein detection and sequencing, contributing to the broader application of nanopore technology in biochemical and clinical research.

## Graphical abstract

Nanopore sensing using the EXP2 nanopore from *Plasmodium falciparum* is enhanced by engineering mutants to reduce electrical noise and improve peptide detection. The EXP2_ΔD231_ mutant showed over 50% noise reduction and successfully discriminated diverse peptides, highlighting its potential for protein sequencing. This advancement broadens nanopore technology's applications in biochemical and clinical research.

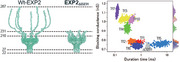

## 1 Introduction

Nanopore sensing has gained significant attention as a technology capable of rapid, label‐free detection of target molecules at the single‐molecule level. This technique involves observing ion flow through nanoscale pores and detecting individual molecules based on changes in ion current as molecules pass through the nanopores. For effective nanopore measurements, it is crucial to select nanopores that are appropriate for the size, charge, and other molecular characteristics of the target molecules. Since the first report in 1996, there have been extensive studies on nanopore detection of single‐stranded DNA,^[^ ^1^, ^2^ ^]^ RNA,^[^ ^2^ ^]^ and peptides,^[^ ^3^ ^]^ molecules with sizes close to the pore diameter of 1.0 nm, using a 1.4 nm diameter α‐hemolysin (αHL) nanopore.^[^ ^4^ ^]^ In 2015, one of the most in‐demand applications, nanopore DNA sequencing, was realized by Oxford Nanopore Technologies, enabling long‐read DNA sequencing.^[^ ^5^, ^6^ ^]^ The potential applications of nanopore sensing have recently expanded to peptide and protein detection and sequencing, and significant research efforts are underway in this direction.^[^ ^7^, ^8^, ^9^, ^10^, ^11^, ^12^, ^13^, ^14^ ^]^

Although many types of nanopores found from natural pore‐forming toxins have been reported (**Table** **1**), pore‐forming transporters have recently emerged for detecting diverse sizes, charges, and structures of peptides and proteins. In particular, translocon, a complex of proteins associated with the translocation of polypeptides across membranes, is a promising candidate for the sensor pore in protein nanopore sequencing due to its very dynamic range of conformational states. For example, Skp and PpiD have been reported to promote translocation by binding to peptide chains emerging from the periplasmic side. In addition, FhuA is conjugated to an antibody‐mimetic protein binder; protein is detected by binding the binder to the target protein. Furthermore, we have reported the polypeptide detection capabilities of the EXP2 nanopore,^[^ ^15^, ^16^ ^]^ a component of the malaria translocon, using channel current measurements.^[^ ^7^ ^]^ The EXP2 nanopore demonstrated the requisite resolution to distinguish the difference in molecular weight of poly‐L‐lysine (PLL) between L‐PLL (molecular weight (*M*_W_): 30 000–70 000) and S‐PLL (*M*_W_: 10 000). The analysis of the detection event frequency indicated that the EXP2 nanopore is more suitable for detecting polycationic peptides than the αHL nanopore. In addition, the inner surface of the alpha‐helical nanopore, such as EXP2 nanopore allows access to more amino acid side chains than beta‐barrel nanopores.^[^ ^17^ ^]^ As the peptide passes through the EXP2 nanopore, it interacts with the side chains of the amino acids on the nanopore surface, which allows it to remain inside the nanopore longer, resulting in improved resolution. To achieve accurate protein sequencing, it is essential to address the issue of gating‐like noise, which appears as intrinsic current noise in biological nanopores, including the EXP2 pore. This noise has a shape similar to that of blocking signals from target molecules, which reduces the accuracy of detection.

**Table 1:** Kinds of Nanopore membrane protein.

Nanopore	Pore diameter [nm]	Target
Alpha‐hemolysin (αHL)	1.4	DNA,^[^ ^25^–^29^ ^]^ RNA,^[^ ^30^ ^]^ PNA,^[^ ^31^ ^]^ metal,^[^ ^32^ ^]^ suger,^[^ ^33^ ^]^ peptide^[^ ^12^, ^34^, ^35^ ^]^
Mycobacterium smegmatis porin A (MspA)	1.2	DNA,^[^ ^36^, ^37^, ^38^, ^39^, ^40^, ^41^, ^42^, ^43^, ^44^, ^45^ ^]^ RNA,^[^ ^45^, ^46^ ^]^ peptide^[^ ^44^, ^47^ ^]^
Aerolysin (AeL)	1.0	DNA,^[^ ^48^, ^49^, ^50^, ^51^ ^]^ peptide,^[^ ^10^, ^11^, ^52^, ^53^, ^54^, ^55^, ^56^ ^]^ polysaccharide^[^ ^57^ ^]^
Outer membrane protein G (OmpG)	1.3	Protein,^[^ ^58^ ^]^ peptide^[^ ^59^ ^]^
Fragaceatoxin C (FraC)	1.6	DNA,^[^ ^60^ ^]^ peptide^[^ ^61^, ^62^, ^63^ ^]^
Gamma‐hemolysin (γHL)	1.6	DNA^[^ ^64^ ^]^
Cytolysin A (ClyA)	3.3	DNA,^[^ ^65^ ^]^ peptide^[^ ^66^, ^67^, ^68^ ^]^
Bacteriophage phi29 (Phi29)	2.8	DNA,^[^ ^69^ ^]^ peptide^[^ ^70^, ^71^ ^]^
CsgG	3.3	DNA^[^ ^5^ ^]^

Nanopore engineering encompasses endeavors to mitigate electrical noise. For example, mutations in the outer membrane protein G (OmpG)^[^ ^18^, ^19^ ^]^ and iota toxin binding component (Ib) nanopores^[^ ^20^, ^21^ ^]^ have been found to significantly reduce noise occurrence,^[^ ^19^, ^22^, ^23^, ^24^ ^]^ thereby enhancing the detection accuracy of molecules such as adenosine diphosphate (ADP).^[^ ^22^ ^]^ The objective of this study is to enhance the detection accuracy by engineering the EXP2 nanopores to eliminate electrical noise. The low‐noise mutants were employed to assess the capability of single amino acid discrimination, and the identification of peptide fragments degraded from target proteins.

## 2 Result and Discussion

### 2.1 Redesign of EXP2 Nanopore for Reducing Gating Noise

**Figure 1:**
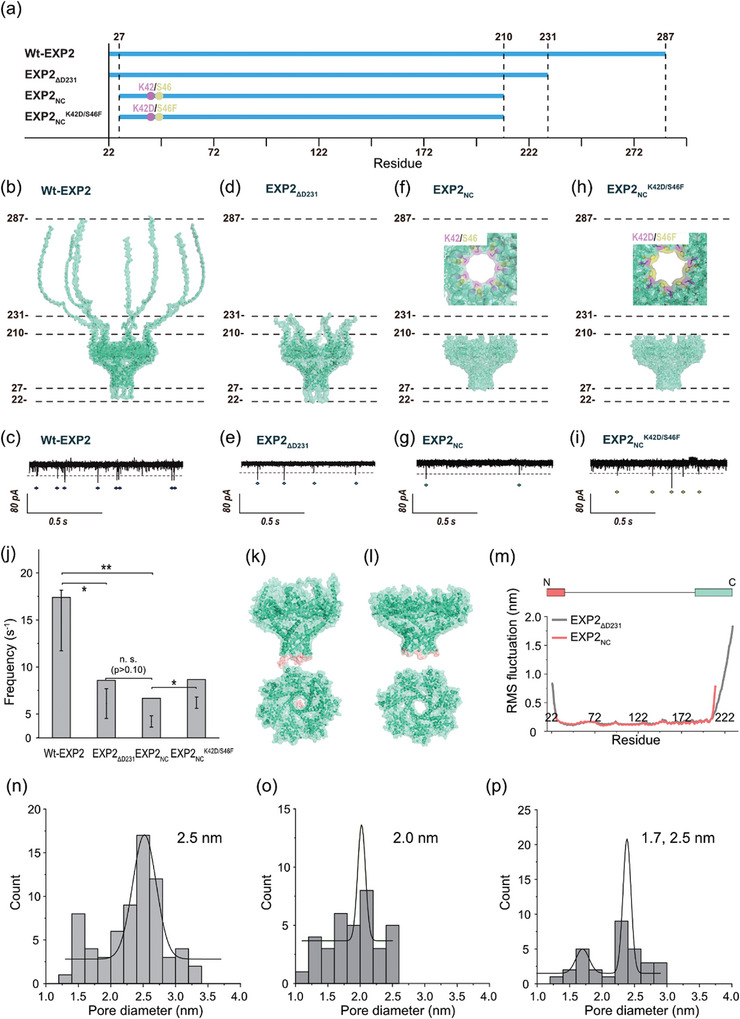
Channel current measurements of EXP2 mutants. a) The sequence, bi) Schematic structure (Top) and typical current–time traces without any additional molecules (Bottom) of b,c) WT‐EXP2 nanopore (*n* = 5), d,e) EXP2_ΔD231_ nanopore (*n* = 69), f,g) EXP2_NC_ nanopore (*n* = 35), and h,i) EXP2_NC_^K42D/S46F^ nanopore (*n* = 32). j) The current noise frequency of the blocking signals of each nanopore. Statistical significance means ^*^: 0.01 < p < 0.05; ^**^: 0.001 < p < 0.01; n. s.: not significant. k,l) Side view (Top) and the top view (Bottom) of the final structure of k) EXP2_ΔD231_ nanopore and l) EXP2_NC_ nanopore. m) Analysis of the RMSFCα of each nanopore. The N‐terminal structure (highlighted in red) of the EXP2_ΔD231_ nanopore has temporarily blocked the interior of the nanopore. MD simulation was conducted on the condition of DOPC membrane, 1 M KCl at 295.15 K. n–p) Histograms of the pore diameter of (n) EXP2_ΔD231_ nanopore, (o) EXP2_NC_ nanopore, and (p) EXP2_NC_^K42D/S46F^ nanopore. These pore diameters were calculated from the ion current conductance using Hille's equation. Data were recording at 22 ± 2 °C and +100 mV in 1 M KCl, 10 mM MOPS, pH 7.0, using a 10 kHz low‐pass Bessel filter with a 50 kHz sampling rate.

In a previous study, we demonstrated that WT‐EXP2 nanopores can distinguish between two types of poly‐L‐lysine: short‐poly‐L‐lysine (S‐PLL, 10 000 Da) and long‐poly‐L‐lysine (L‐PLL, 30 000–70 000 Da).^[^ ^7^ ^]^ This distinction was based on the current blockings observed. Two significant challenges must be overcome to extend peptide detection and identification to more complex sequences. The first challenge is the presence of gating‐like noise signals originating from WT‐EXP2 nanopores, which can make it difficult to discriminate between the blocking signals caused by target molecules (**Figure** **1**a–c).^[^ ^7^ ^]^ The second challenge is improving the detection resolution. In our previous study, despite the 20 000 Da difference in molecular weight between the two short and long PLLs, it was necessary to perform statistical analysis in order to distinguish between them based on the raw data. This low resolution will facilitate the identification of peptides with smaller molecular weight differences.

To enhance the accuracy of peptide identification, we initiated the engineering and creation of 3 mutants of translocon EXP2 (Figure 1a,d–i). The EXP2 protein is a transmembrane component of the *Plasmodium* translocon of exported proteins (PTEX)^[^ ^16^, ^74^, ^75^ ^]^ It contains binding motifs that enable its assembly with other PTEX units. It is postulated that the assembly strand (D231–E287) in EXP2 is capable of movement, which may result in temporarily blocking the nanopore and generation of gating noise. To substantiate this hypothesis, a mutant EXP2_ΔD231_ nanopore, in which the assembly strand has been deleted (Figure 1d; Table S1, Supporting Information), was expressed. Following protein expression and purification in *E. coli*, SDS‐PAGE demonstrated the presence of a band with a mass corresponding to that of the EXP2_ΔD231_ monomer (24.5 kDa; Figure S1a, Supporting Information). Furthermore, the channel current measurements provided evidence that the mutant had formed pores, as illustrated in Figure 1e. Regarding the noise characteristics of the EXP2_ΔD231_ mutant, the frequency of the noise was demonstrably reduced by approximately 50%, indicating that the significant noise was attributable to the assembly strands (Figure 1e,j). Nevertheless, gating‐like noise signals persist in this mutant. To ascertain the source of these electrical noise phenomena, molecular dynamics (MD) simulations of EXP2_ΔD231_ and EXP2_NC_ were conducted. The simulations were conducted under analogous conditions to those employed in the nanopore measurements, including a 2,3‐Dioleoyl‐glycero‐1‐phosphocholine (DOPC) membrane, 1 M KCl, and 295.15 K. Typical snapshots are presented in Figure 1k,l. The root means square fluctuation (RMSF) of the C‐terminal amino acids in both EXP2_ΔD231_ and EXP2_NC_ was found to be significant, and it was therefore deemed reasonable to remove this region in order to reduce the noise (Figure 1j). As observed in the RMSF of the N‐terminal region (V22–27), this region appeared to move and temporarily block the pore (Figure 1k,m), which likely contributed to the electrical noise.

Based on the aforementioned results, another mutant, EXP2_NC_, was designed by deleting the N‐ and C‐terminal regions (N: ≈Y28, C: ≈E211; Table S1, Supporting Information). The mutant was successfully expressed (Figure S1b, Supporting Information) and exhibited a pore configuration with significantly reduced noise, demonstrating a 70% reduction compared to the WT‐EXP2 (Figure 1g,j). This reduction was also corroborated by MD simulation, wherein the RMSF value in both the N‐ and C‐terminal regions was nearly undetectable (Figure 1m). However, gating noise persisted, potentially attributable to hydrophobic gating of the pore, which was not captured in our 200 ns MD simulation.^[^ ^76^ ^]^

### 2.2 Redesign of EXP2 Nanopore for the Peptide Fragments Detection

We proceed to construct a mutant of EXP2_NC_ with the lowest electrical noise to detect cationic peptides. This mutant is designed to exhibit high frequency and accuracy for target detection. In principle, the target molecule is captured in the pore using a combination of electrophoretic force (EPF) and electroosmotic flow (EOF). The EPF is dependent on the applied voltage and the charge of the target molecule, while the EOF is dependent on the surface charge and ions inside the pore. Estimating the EPF based on the applied voltage and target charge was relatively straightforward; however, the EOF was more challenging. To investigate the strength and direction of the EOF, MD simulations were performed.

An EOF is typically contingent upon the charge density within the interior pore. It was anticipated that the EOF and EPF would exhibit a similar directionality, from the *cis* to *trans* side, due to the presence of a negative pore wall in the EXP2. However, the MD simulation indicated that the EOF direction was contrary to the EPF (Figure S2a, Supporting Information), indicating that the charge at the entrance (K42) significantly influences the EOF and competes with the EPF (Figure S2c, Supporting Information). Consequently, a mutation was introduced at residue K42 with the objective of facilitating a synergistic interaction between the EOF and EPF. Furthermore, to improve the detection precision of peptide fragments, we substituted the amino acid S46 with an aromatic amino acid (F) within the nanopore, situated at a distance of 1.4 nm from the negatively charged amino acid in the restriction site, in accordance with the findings of previous studies.^[^ ^77^ ^]^ The aforementioned mutations were achieved through two mechanisms: 1) electrostatic interaction between the negatively charged restriction and the target cationic peptide, which was induced by K42D, and 2) cation‐π interaction between the aromatic amino acid and the target cationic peptide (S46F). Prior to the expression of the mutant, the effects of the mutation on EOF were analyzed using MD simulation. The results indicated that the direction of EOF after mutation (K42D/S46F) was aligned with the direction of EPF (Figure S2b, Supporting Information), and the strength of EOF was found to have increased by approximately 10‐fold (Figure S2c, Supporting Information). Consequently, an attempt was made to create a mutant EXP2_NC_^K42D/S46F^ nanopore (Figure 1h; Table S1, Supporting Information).

The EXP2_NC_^K42D/S46F^ was expressed by *E. coli* and formed a stable nanopore structure, as evidenced by nanopore recordings (Figure 1i). With regard to the gating noise of this mutant, the frequency of the noise signal's appearance was slightly higher than that of EXP2_NC_, while it was almost identical to that of EXP2_ΔD231_ (Figure 1i). It is noteworthy that the blocking time of gating noise in mutants lacking the C‐terminal region (EXP2_NC_ and EXP2_NC_^K42D/S46F^) was considerably longer than that observed in the wild‐type (WT) and EXP2_ΔD231_ strains, as illustrated in Figures S3 and S4 (Supporting Information). This finding indicates the existence of a distinctive gating mechanism, which is hypothesized to be caused by an increased hydrophobicity of the nanopore surfaces, leading to hydrophobic gating. Subsequently, the pore diameters of the EXP2 mutants were calculated using Hille equation. All three mutants, EXP2_ΔD231_, EXP2_NC_, and EXP2_NC_^K42D/S46F^, have a pore diameter of approximately 2.0–2.5 nm, as calculated from the conductance data (Figure 1n–p). The EXP2_NC_^K42D/S46F^ exhibits a second and smaller pore with a diameter of approximately 1.7 nm (Figure 1p). The formation of two nanopores of varying sizes was contingent upon the number of monomers, a phenomenon determined by the distinct conformations of the nanopore complex. This result lends further support to the hypothesis that EXP2_NC_^K42D/S46F^ exhibits significant pore fluctuations. The pore diameters were estimated through the use of HOLE analysis,^[^ ^73^ ^]^ as illustrated in Figure S5 (Supporting Information). The EXP2_NC_^K42D/S46F^ nanopore has a larger pore (Figure S5c, Supporting Information) and exhibits a more substantial structural alteration in comparison to the EXP2_NC_ nanopores (Figure S5d, Supporting Information). In light of these findings, we postulate that the alteration in the nanopore configuration is the underlying cause of the electrical noise observed in EXP2_NC_^K42D/S46F^. It is noted that the discernible behavior extends beyond 200 ns in molecular dynamics simulations, rendering the structural fluctuations observed in this study inconclusive concerning their origin.

### 2.3 Capability of Single Amino Acid Identification using EXP2 Mutants

**Figure 2:**
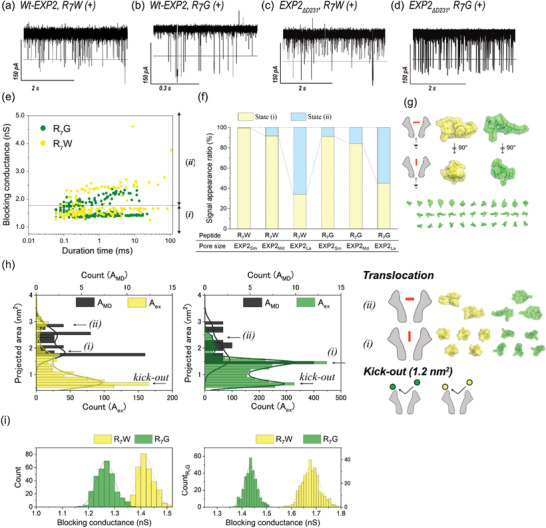
Oligo‐arginine peptides detection using Wt‐EXP2 nanopore and EXP2_ΔD231_ nanopore. a–d) Typical current–time traces with 500 nM of R_7_W and R_7_G using Wt‐EXP2 nanopore (a,b, R_7_G: n = 228, R_7_W: n = 217), EXP2_ΔD231_ nanopore (c,d, R_7_G: n = 298, R_7_W: n = 293). e) Scatter plot of duration time versus current blocking conductance of the EXP2_ΔD231_ nanopore with R_7_W (yellow), and R_7_G (green). f) Signal appearance ratio of state (i) and state (ii) for different sizes of EXP2_ΔD231_ nanopore. g) The top cluster structure of R_7_X was obtained from 100 ns MD simulation. Each structure reflects the peptide shape seen from inside the nanopore. The top is the shape when the peptide passes through the nanopore from the lateral direction, and the bottom is the shape when it passes through the nanopore from the vertical direction. h) The histogram of the projected area from the blocking conductance and the MD simulation of R_7_W (left), and R_7_G (right). The p‐values between *A_MD_* and *A_ex_* excluding kick‐out was more than 0.10. i) Comparison of the blocking conductance histograms of the current noise of each nanopore (gray), R_7_W (yellow), and R_7_G (green) using Wt‐EXP2 nanopore (left), and EXP2_ΔD231_ nanopore (right). Data were recorded at 22 ± 2 °C and +100 mV in 1 M KCl, 10 mM MOPS, pH 7.0, using a 10 kHz low‐pass Bessel filter with a 50 kHz sampling.

The WT‐EXP2 assay confirmed the presence of cationic poly‐L‐lysine, as previously reported in our research.^[^ ^7^ ^]^ In this study, we evaluate the ability of EXP2 mutants to discriminate between single amino acids. We used hepta‐arginine (R7) in combination with various amino acids (X)^[^ ^10^ ^]^ for this experiment. The cationic peptides, R_7_X, were captured by nanopores based on electrophoretic principles.^[^ ^78^ ^]^ Among the R_7_X peptides, R_7_W (containing a tryptophan residue with the largest side chain, molecular mass: 1297 Da) and R_7_G (containing a glycine residue with the smallest side chain, molecular mass: 1168 Da) were specifically examined. The channel recordings with all EXP2 nanopores demonstrated current blocking during the translocation for both peptides, R_7_W and R_7_G (**Figure** **2**a–d). Initially, the noise issue was investigated. It was observed that the detection frequency of R_7_X peptides was higher than the frequency of electrical noise originating from the nanopore (Figure S6, Supporting Information). Furthermore, the three types of EXP2 mutants exhibited a higher signal‐to‐noise ratio.

Figure 2e depicts the scatter plot of the blocking current and duration for R_7_G and R_7_W, as detected by EXP2_ΔD231_ pore. Two distinct states in the blocking conductance were observed for both R_7_W and R_7_G measurements, and the conductance range could be divided into two regions: (i)blocking conductance ≤ 1.75 nS, (ii) blocking conductance ≥ 1.75 nS (Figure 2e,f). These two conductance states, observed for the same molecule, are likely due to variations in the orientation of peptide entry into the nanopore. For linear peptides such as R_7_X, the projected area within the nanopore changes depending on whether the entry is vertical or horizontal, leading to variations in the blocking conductance. This will result in a change in the quantity of the blocking conductance. To test this hypothesis, we compared the projected area estimated from MD simulations with the experimentally determined projected area from the blocking conductance. To estimate the molecular structure of R_7_X, AlphaFold^[^ ^79^, ^80^ ^]^ was employed. Subsequently, molecular dynamics simulations were conducted using the aforementioned structure under identical experimental conditions (DOPC membrane, 1 M KCl, 295.15 K) to determine its most probable conformation. Subsequently, the structure was rotated by 30° in each of the x, y, and z directions to estimate the potential projected areas of R_7_X (Figure 2g). The following formula was employed to estimate the projected area (*A*_ex_) from the blocking conductance:

(1) $$\mathrm{Projected}\;\mathrm{area}\;\left(\mathrm{Aex}\right)=\frac{G_{\mathrm{block}}\left(x\right)\cdot S}{G_{\mathrm{open}}}$$

where *G*_block_ represents the blocking conductance for each signal, *S* is the pore area during open state calculated from the diameter of the formed nanopore, and *G*_open_ is the open‐state conductance. A comparison of the estimated and experimentally measured projected areas revealed that the range of estimated projected areas (*A*_MD_) for R_7_W and R_7_G was found to closely match the range of experimentally determined values (*A*_ex_) (Figure 2h). There was no statistically significant difference between *A*_MD_ and *A*_ex_ excluding kick‐out. Moreover, two peaks were identified in *A*_ex_, suggesting the presence of vertically or horizontally oriented entrances. In the subsequent analysis, a projected area smaller than approximately 1.2 nm^2^ was classified as being kicked out (not translocating the nanopore and returning to the original solution) due to the minimum estimated value of *A*_MD_ for R_7_G being 1.5 nm^2^ ± 0.1.

The peak difference in the blocking conductance between R_7_W and R_7_G was 0.15 nS when WT‐EXP2 nanopore was used (Figure 2i, left). The conductance values for R_7_W and R_7_G were 1.41 nS and 1.26 nS, respectively. In contrast, the use of EXP2_ΔD231_ nanopores resulted in a notable expansion of the peak difference, reaching 0.25 nS (R_7_W: 1.68 nS, R_7_G: 1.43 nS, as illustrated in Figure 2i, right). The blocking conductance in WT‐EXP2 was observed to be smaller than that in EXP2_ΔD231_. This discrepancy is likely attributable to the presence of considerable noise signals within the WT‐EXP2 system. The blocking conductance value obtained in the EXP2_ΔD231_ measurement was consistent with the calculated value from *A*_MD_, indicating that the peptide entered the nanopore vertically. Although the higher capture frequency was exhibited by EXP2_NC_ and EXP2_NC_^K42D/S46F^ (Figure S6, Supporting Information). Compared to EXP2_ΔD231_, the EXP2_NC_ series has a higher proportion of forming small pores, so the peptides insertion from the vertical direction has increased. When the main direction of the peptide passes through the nanopore from the vertical direction, it becomes difficult to distinguish the peptide because the difference in the projected peptide is small. Consequently, the employment of the EXP2_ΔD231_ nanopore enabled the precise reflection of peptide‐derived blocking conductance, thereby enhancing discrimination capability.

### 2.4 Peptide Fragments Detection using EXP2_ΔD231_ Nanopore

Next, we employed the EXP2_ΔD231_ nanopore, which exhibited augmented discrimination capabilities, with the objective of detecting peptide fragments and thereby identify the original protein within the nanopore proteome. This study employed 8 types of trypsin‐digested peptides (Tf: trypsinized fragments) from lysozyme, as previously reported.^[^ ^77^, ^81^, ^82^ ^]^ In these reports, 10 types of Tf peptides (molecular weight ranging from 517.3 to 2508 Da, in ascending order from Tf1 to Tf10) were measured using biological nanopores, including Fragaceatoxin C (FraC), Aerolysin (AeL), and Cytolysin K (CytK). Notably, the molecular weight of the Tf peptides that could be detected exhibited variability contingent on the specific nanopore utilized. It was observed that FraC could detect Tf4 to Tf7,^[^ ^82^ ^]^ while AeL and CytK were observed to detect Tf2 to Tf9.^[^ ^77^, ^81^ ^]^ The simultaneous detection of Tf1 (molecular mass: 517.3 Da) and Tf10 (molecular mass: 2508 Da) using a single nanopore represented a significant challenge. Moreover, utilizing a single nanopore type to detect peptides across the entire molecular mass range of Tf1 to Tf10 has also proven to be a considerable obstacle.

**Figure 3:**
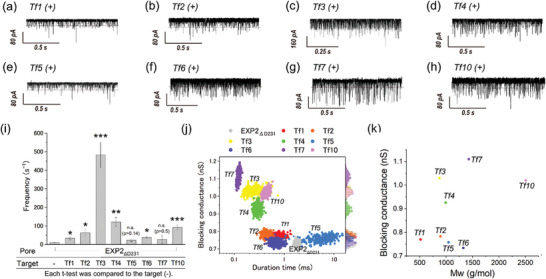
Tf peptides detection using EXP2_ΔD231_ nanopore. The amino acid sequence of synthetic trypsinized fragments from lysozyme including their mass, and total charge at pH 7.0 and pH 3.8. C_m_ means an alkylation of the cysteine residues. a–h) Typical current–time traces of EXP2_ΔD231_ with 50 µM of (a) Tf1 (*n* = 3818), (b) Tf2 (*n* = 2241), (c) Tf3 (*n* = 7265), (d) Tf4 (*n* = 1074), (e) Tf5 (*n* = 929), (f) Tf6 (*n* = 4053), (g) Tf7 (*n* = 478), (h) Tf10 (*n* = 1975) peptide. i) The event frequency of the blocking signals reflecting the detection of individual Tf peptides. Statistical significance is shown between current noise of EXP2_ΔD231_ and each Tf peptide (^*^: 0.01 < *p* < 0.05; ^**^: 0.001 < *p* < 0.01; ^***^: *p* < 0.001, n. s.: not significant). j) Scatter plot of duration time versus current blocking conductance. The bootstrapped data were used to produce the scatter plot (Tf1: 0.77 nS, Tf2: 0.78 nS, Tf3: 1.0 nS, Tf4: 0.92 nS, Tf5: 0.75 nS, Tf6: 0.734 nS, Tf7: 1.1 nS, Tf10: 1.02 nS). k) Relationship between the molecular mass of peptides and the blocking conductance at pH 3.8. No error bars due to bootstrapping of the blocking conductance. Each color of the plot reflects the Tf peptides. Data were recorded at 22 ± 2 °C and +100 mV in 1 M KCl, 10 mM citric acid, pH 3.8, using a 10 kHz low‐pass Bessel filter with a 50 kHz sampling rate.

Using the EXP2_ΔD231_ nanopore, we endeavored to detect from Tf1 to Tf7 and Tf10 under pH 3.8 as the same solution condition of the previous reports (Table S2, Supporting Information). WT‐EXP2 and its mutants including EXP2_ΔD231_ form a nanopore with a wide range of conductance (Figure 1m), and this enables the detection of small (Tf1) to large (Tf10) peptides (**Figure** **3**a–h). The projected area histograms calculated from the experiment of each Tf peptides using EXP2_ΔD231_ nanopore (Figure S7, Supporting Information) also exhibited two distinct conductance distributions. Since the lower limit of the projected area for Tf peptides (Tf1) was 0.6 nm^2^, blocking signals calculated with larger than 0.6 nm^2^ were used in the analysis. Regarding the frequency of the blocking signals, Tf3 (net charge: +2) was found to have the highest frequency, while Tf5 (net charge: +0.7) had the lowest frequency (Figure 3i). Generally, molecules are captured by the nanopore due to both the EPF generated by the molecular charge and the EOF generated by the internal charge of the nanopore.^[^ ^55^, ^78^, ^84^, ^85^, ^86^ ^]^ The results are consistent with the charge states of the Tf peptides under the experimental conditions at pH 3.8. Further analysis of the blocking duration and conductance led to successful discrimination among the 8 types of Tf peptides, Tf1 to Tf7 and Tf10 (Figure 3j). The blocking conductance of four peptides was consistent with molecular weight: Tf7 > Tf4 > Tf2 > Tf1 (Figure 3k), but the conductance for Tf5, Tf6, and Tf10 was lower than expected based on their molecular weight, while the value for Tf3 was unusually high (Figure 3j,k). For Tf5, Tf6, and Tf10, the experimental projected area, *A*_ex_, was lower than the *A*_MD_, suggesting that Tf peptides were ejected from the pore without translocation, as shown in Figure S7e,f,h (Supporting Information). For Tf3, a notable number of passages were observed with the peptide oriented horizontally (Figure S7c, Supporting Information), likely due to the charged position that Tf3 has a charge of +2, with its positive charges located at both ends (Table S2, Supporting Information). The EPF acting at both ends of Tf3 likely disrupts a consistent orientation for passage through the nanopore, resulting in a higher incidence of horizontal entry. This orientation variability is thought to contribute to the relatively higher blocking conductance observed for Tf3. The EXP2_ΔD231_ nanopore effectively detects a wide range of peptide fragments, showing improved sensitivity and versatility over other nanopores. While the simultaneous detection of both the smallest and largest peptides remains a significant challenge, the ability to identify the smaller peptide could prove invaluable in facilitating the comprehensive identification of proteins and advancing the field of nanopore proteomics.

## 3 Conclusion

In this study, we attempted to improve the molecular detection precision of the EXP2 nanopore for peptide sensing by engineering several mutants, including EXP2_ΔD231_, EXP2_NC_, and EXP2_NC_^K42D/S46F^. The EXP2_ΔD231_ mutant significantly reduced the electrical noise by more than 50%, enhancing the discrimination accuracy of peptide detection, specifically for two oligoarginine peptides. Furthermore, the EXP2_ΔD231_ nanopore demonstrated the capability to detect and discriminate a broad range of peptides, from small peptides like Tf1 to larger peptides like Tf10, within a single nanopore. This capability extends beyond the detection range typically achieved with other nanopore types, which have made significant progress in peptide detection.

The findings also revealed a correlation between the projected area of peptides during nanopore translocation and their blocking conductance, suggesting potential applications of nanopore measurements for structural analysis of peptides. The successful detection of peptides with the redesigned EXP2 nanopores holds significant promise for the future development of nanopore‐based technologies for nanopore proteome and sequencing. Our study provides valuable insights and a foundational basis for expanding the application of nanopore technology in peptide detection and characterization.

## 4 Experimental Section

### Regents and Chemicals

The reagents used in this study were as follows: KOD SYBR qPCR Mix (TOYOBO Co., Ltd., Japan) and NucleoSpin Gel and PCR Clean‐up (Takara Bio Inc., Japan) were used for DNA amplification. The pGEX‐3X vector (GE Healthcare, UK), In fusion Snap Assembly Master Mix (Takara Bio Inc., Japan), and PrimeSTAR MAX Mutagenesis Basal Kit (Takara Bio Inc., Japan) were used in the gene cloning. In the EXP2 protein expression and purification experiments, *E.col* BL21 (DE3) competent call (SMOBIO Technology, Inc., Taiwan), sodium chloride (NaCl; FUJIFILM Wako Pure Chemical Corporation, Japan), potassium chloride (KCl; FUJIFILM Wako Pure Chemical Corporation, Japan), disodium hydrogen phosphate (Na_2_HPO_4_; FUJIFILM Wako Pure Chemical Corporation, Japan), potassium dihydrogen phosphate (KH_2_PO_4_; NACALAI TESQUE, Inc., Japan), 2‐Amino‐2‐hydroxymethyl‐propane‐1,3‐diol (Tris‐HCl; FUJIFILM Wako Pure Chemical Corporation, Japan), reduced glutathione (Cytiva, Japan), and dithiothreitol (DTT; FUJIFILM Wako Pure Chemical Corporation, Japan) were used for buffer solutions of the protein purification.

1, 2‐diphytanoyl‐*sn*‐glycero‐3‐phosphocholine (DPhPC; Avanti. Polar Lipids, Inc., USA), *n*‐decane (Wako Pure Chemical Industries, Ltd., Japan), potassium chloride (KCl; Nacalai Tesque, Inc., Japan), 3‐morpholinopropane‐1‐sulfonic acid (MOPS; Nacalai Tesque, Inc., Japan). Buffered electrolyte solutions (1 M KCl, 10 mM MOPS at pH 7.0, and 1 M KCl, 10 mM citric acid at pH 3.8) were prepared using ultrapure water, which was obtained from a Milli‐Q system (Millipore, Billerica, MA, USA). Wild‐type alpha‐hemolysin (αHL; Sigma‐Aldrich, St. Louis, MO, USA, and List Biological Laboratories, Campbell, CA, USA) was obtained as the monomer polypeptide, isolated from *Staphylococcus aureus* in the form of a powder and dissolved at a concentration of 1 mg mL^−1^ in ultrapure water. For use, samples were diluted to the designated concentration using a buffered electrolyte solution and stored at 4 °C. The oligo‐arginine peptides (R_7_X, X = G, W), and trypsinized fragments from lysozyme (Tf1–Tf7, Tf10) were synthesized by Greiner Bio‐One Co. Ltd (Tokyo, Japan) and dissolved at a concentration of 1 mM in ultrapure water respectively, stored at −20 °C.

### Cloning of the EXP2 Gene from *P. falciparum* 3D7

The production of wild‐type EXP2 (WT‐EXP2) was carried out referring to our previous research.^[^ ^15^ ^]^ The full‐length EXP2 gene from *P. falciparum* 3D7 (PF14_0678) was amplified by PCR and inserted into the *Bam*HI/*Eco*RI site of the pGEX‐3X plasmid. EXP2 gene includes glutathione S‐transferase tag (GST‐tag) at the N‐terminus of EXP2, and a human rhinovirus (HRV) 3C protease recognition site (Leu‐Glu‐Val‐Leu‐Phe‐Gln‐Gly‐Pro) between GST and EXP2. Based on the pGEX‐3X‐HRV3C‐EXP2 plasmid, the EXP2 genes of each mutant (EXP2_ΔD231_, EXP2_NC_, EXP2_NC_^K42D/S46F^) were amplified by PCR. The amplified PCR products were inserted into the EXP2 gene site of the pGEX‐3X‐HRV3C‐EXP2 plasmid using an In‐fusion HD Cloning Kit (Takara Bio Inc., Japan), and a Quick‐change Site‐Directed Mutagenesis Kit (Agilent Technologies Japan, Ltd., Japan).

### Expression and Purification of EXP2 Mutants

The constructed plasmids were transformed into chemically competent *Escherichia coli* BL21 (DE3) cells with ampicillin selection. The transformed cells were grown in Lysogeny broth with 100 µg mL^−1^ ampicillin at 37 °C until the cells reached an OD_600_ of 0.4–0.6. Protein production was induced with 1 mM Isopropyl‐β‐D‐thiogalactopyranoside (IPTG), and shaken (70 rpm) for overnight, at 18 °C. Afterward, the cells were harvested by centrifugation for 15 min at 5000 rpm, and the collected protein was stored at −20 °C.

The lysate was suspended with buffer A (phosphate‐buffered saline (PBS), 137 mM NaCl, 2.7 mM KCl, 10 mM Na_2_HPO_4_, 1.8 mM KH_2_PO_4_, pH 7.4) with 1 mM dithiothreitol (DTT). The cells were disrupted by sonication using a UD‐211 ultrasonic disruptor (TOMY DIGITAL BIOLOGY CO., LTD., Japan), and the EXP2 protein was collected by centrifugation for 20 min at 14 000 g and filtered through a membrane with 0.45 µm pores (ADVANTEC TOYO KAISHA, LTD., Japan). After syringe filtering, the supernatant was incubated with 0.05% *n*‐dodecyl‐β‐d‐maltopyranoside (DDM) (Dojindo Laboratories, Japan) to prevent the aggregation of EXP2. The supernatant containing DDM was loaded to a Glutathione Sepharose 4B column (GE Healthcare, Japan). The column was washed five times using buffer A (PBS with 0.02% DDM). After alternating the buffer solution in the column from buffer A to buffer B (50 mM Tris‐HCl, 200 mM NaCl, 0.02% DDM, pH 8.0), HRV 3C protease (Funakoshi Co., Ltd., Japan) in buffer B was applied to the column, and the column was incubated at 4 °C for overnight to facilitate the proteolysis. After the elution, protein concentrations were determined by measuring the absorbance at 280 nm using a NanoDrop 2000 spectrophotometer (ThermoFisher Scientific, USA). The EXP2 monomers were dissolved in 20% glycerol and stored at −80 °C.

### SDS‐Polyacrylamide Gel Electrophoresis (PAGE)

Each EXP2 mutant was mixed with sample buffer at a volume ratio of 1:1. These samples were electrophoresed on a 12–20% sodium dodecyl sulfate‐polyacrylamide gel (SDS‐PAGE). The electrophoresis was performed at 25–35 mA by using NC‐1017 (NIHON EIDO Co., LTD., Japan), and the gel was stained with Quick‐CBB (FUJIFILM Wako Pure Chemical Corporation, Japan).

### Fabrication of the Microdevice

The device‐body and separator were cut from polymethyl methacrylate (PMMA, the device‐body: 6 mm thick, separator: 0.2 mm thick) plate (Mitsubishi Rayon, Tokyo, Japan) with a 3D modeling machine (MM‐100, Modia Systems, Japan). Two wells (2.0 mm diameter and 4.5 mm depth) and a chase between the wells were manufactured on the device‐body. Each well had a through‐hole in the bottom and Ag/AgCl electrodes were set into this hole. A polymeric film made of parylene C (polychloro‐p‐xylylene) with a thickness of 5 µm was patterned with a single pore (100 µm diameter) using a conventional photolithography method and then fixed between separators using an adhesive bond (Super X, Cemedine Co., Ltd., Tokyo, Japan). The films, including the parylene film, were inserted into the chase to separate the wells.

### Lipid Bilayer Preparation and Reconstitution of the EXP2 Nanopore

Lipid bilayers were prepared into the micro‐device fabricated by microfabrication. Lipid bilayers can be simultaneously formed in this device by the droplet contact method.:^[^ ^72^ ^]^ each chamber of the device was filled with n‐decane (0.5 µL) containing the lipid composition of DPhPC (10 mg mL^−1^). The buffer solution (4.7 µL) containing EXP2 (final concentration 80 nM) was poured into the grounded chamber. The buffer solution (4.7 µL) was also poured into the recoding chamber. Within a few minutes, lipid bilayers formed and EXP2 created nanopores by reconstitution in the lipid bilayers. If the lipid bilayers ruptured during this process, they were recreated by tracing at the interface of the droplet with a hydrophobic stick.

### Channel Current Measurement and Data Analysis

The channel current was recorded using an Axonpatch 200B amplifier (Molecular Devices, USA) or a Pico patch‐clamp amplifier (Tecella, Foothill Ranch, CA, USA). The recorded data from Axonpatch 200B were acquired with Clampex 9.0 software (Molecular Devices, USA) through a Digidata 1440A, and Digidata 1550B analog‐to‐digital converter (Molecular Devices, USA). The sampling frequency was 50 kHz with a 10 kHz low‐pass Bessel filter in Axonpatch 200B at 22 ± 2 °C. The current data was analyzed with Clampfit 11.2 (Molecular Devices, USA), Excel (Microsoft, Washington, USA), Origin pro 2022b (Light Stone, Tokyo, Japan), and Python 3.5 (Python Software Foundation, USA). The *t*‐test was used to test for significant differences.

### Detection of R_7_X Peptides

To insert R_7_X into the outer membrane region of the nanopore, the recording‐side solution contained R_7_X along with either the EXP2 or αHL nanopore. The insertion direction is expected to proceed from the recording side. The recoding side solution contained R_7_X, and EXP2 or αHL nanopore. A constant voltage of +100 mV was applied to the recording chamber.

### Detection of Tf Peptides

The recoding side solution contained Tf peptides, and the grounded side solution contained EXP2. A constant voltage of +100 mV was applied to the recording chamber.

### Estimation of the Pore Properties via Molecular Dynamics (MD) Simulations

The initial structure of wild‐type EXP2 oligomer was taken from the Protein Data Bank (PDB ID: 6e10). To prepare the structure of mutants, the terminus region and introduced the amino acid mutation in the constriction region (K42D/S46F) based on wild‐type EXP2. CHARMM‐GUI was used to construct the initial structures of EXP2 mutants was deleted. All simulation was conducted using GROMACS with the CHARMM36m force field. The simulation for 100–200 ns with 1 M KCl was conducted at the temperature range from 295.15 to 375.15 K. The resulting structures were visualized with PyMOL, and visual molecular dynamics (VMD). Pore diameter was analyzed using HOLE software.^[^ ^73^ ^]^ The RMSFs of the EXP2 mutants and the projected areas of R_7_X were calculated using data from the entire simulation time. All simulation boxes in which the EXP2 mutant detected peptides were set so that the applied voltage of the entire system was 150 mV.

## Conflict of Interest

The authors declare no conflict of interest.

## Author Contributions

M.M. and M.Y. contributed equally to this work. M.M. and R.K. conceived the project and designed the experiments. M.M. performed the experiments and collected the data. M.M. and M.Y. analyzed the data. N.K. and M.Y. synthesized and confirmed the structure of EXP2 and these mutants. The manuscript was written with contributions from all authors.

## Supporting information



Supporting Information

## Data Availability

The data that support the findings of this study are available from the corresponding author upon reasonable request.
