# Supplementary material for: Redesign of Translocon EXP2 Nanopore for Detecting Peptide Fragments
Source: Small Methods. 2025 Feb 5;9(4):2401562. doi: 10.1002/smtd.202401562 (PMC12020339; doi:10.1002/smtd.202401562)
Supplement: Supplementary file 1 — Supporting Information [file SMTD-9-2401562-s001.docx]

Supporting Information

**Redesign of Translocon EXP2 Nanopore for Detecting Peptide Fragments**

*Mitsuki Miyagi^†^, Misa Yamaji^†^, Nina Kurokawa, Masafumi Yohda, Ryuji Kawano**

*Department of Biotechnology and Life Science, Tokyo University of Agriculture and Technology, Tokyo, 184-8588, Japan*

*Corresponding author: rjkawano@cc.tuat.ac.jp

^†^ Equal contribution

Keywords: nanopore, peptide sensing, lipid bilayer, translocon, microfluidics


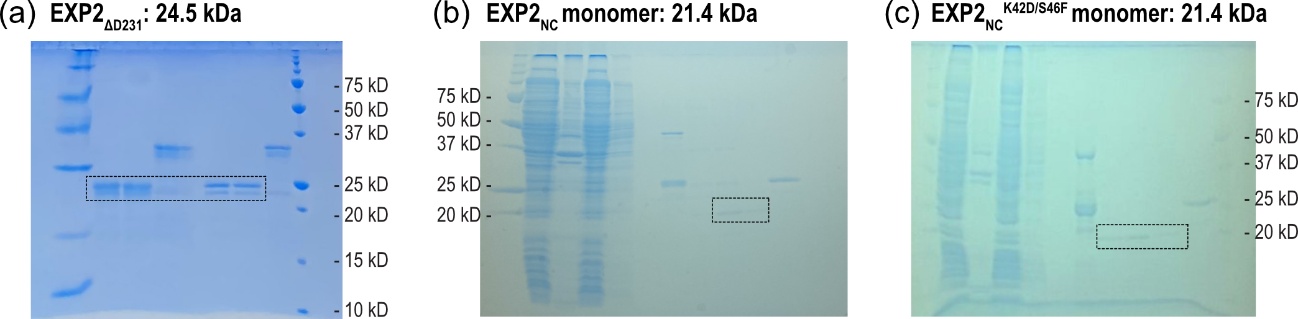
**Figure S1.** SDS-PAGE of EXP2 mutants. The purified each mutant (0.02% DDM) was mixed with an equal volume of the sample buffer and then subjected to SDS-PAGE. Each band surrounded by black dashed line indicates **(a)** EXP2_ΔD231_ monomer, **(b)** EXP2_NC_ monomer, **(c)** EXP2_NC_^K42D/S46F^ monomer.

**
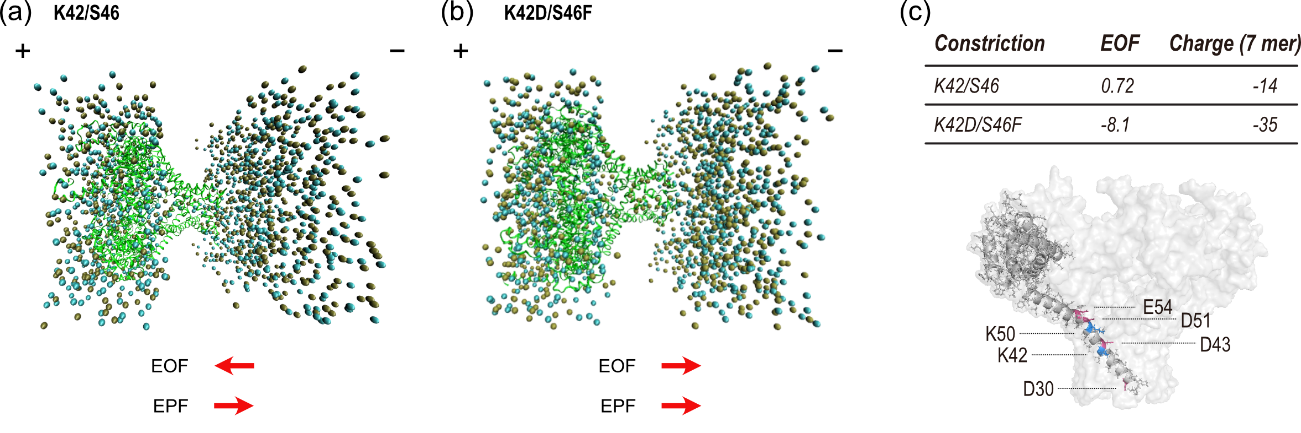
Figure S2.** EOF calculation of the EXP2 nanopore using MD simulation. **(a-b)** Side view of ions (gold: K^+^, blue: Cl^-^) passing through the EXP2_ΔD231_ nanopore with the constriction region of **(a)** K42/S46, and **(b)** K42D/S46F. **(c)** (Top) The direction and strength of the EOF, and charge of the transmembrane domain of each nanopore. (Bottom) Charge distribution of the EXP2 nanopore.

**
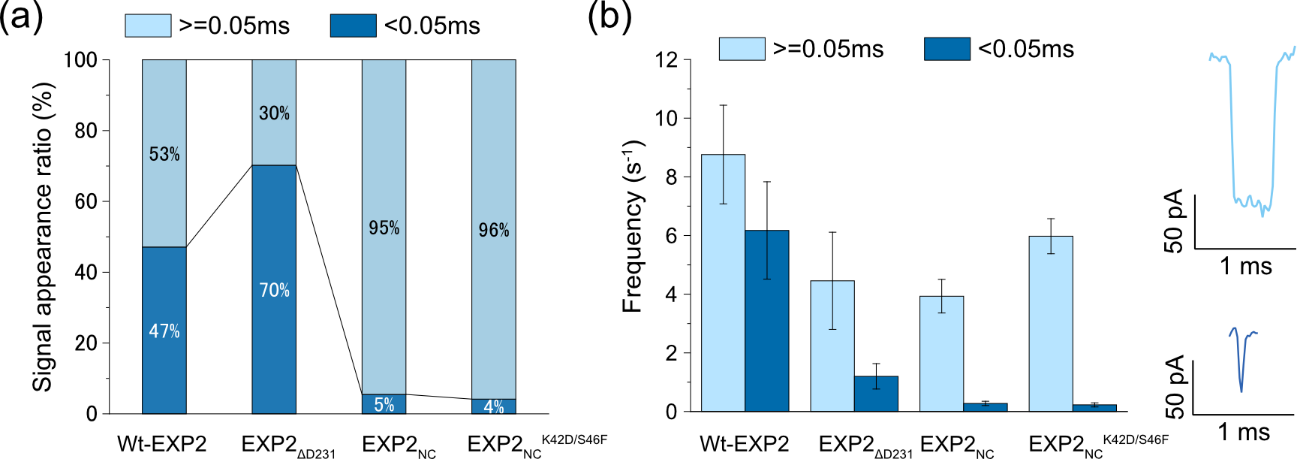
Figure S3.** The current noise properties of WT-EXP2 and its mutants. **(a)** The appearance ratio of the duration time above and below 0.05 ms of the current noise of each nanopore. **(b)** The appearance frequencies of the current noise of nanopore above and below 0.05 ms of the duration time. The appearance ratio and the frequencies of the duration time less than 0.05 ms was considerably decreased by using EXP2_NC_, and EXP2_NC_^K42D/S46F^.

**
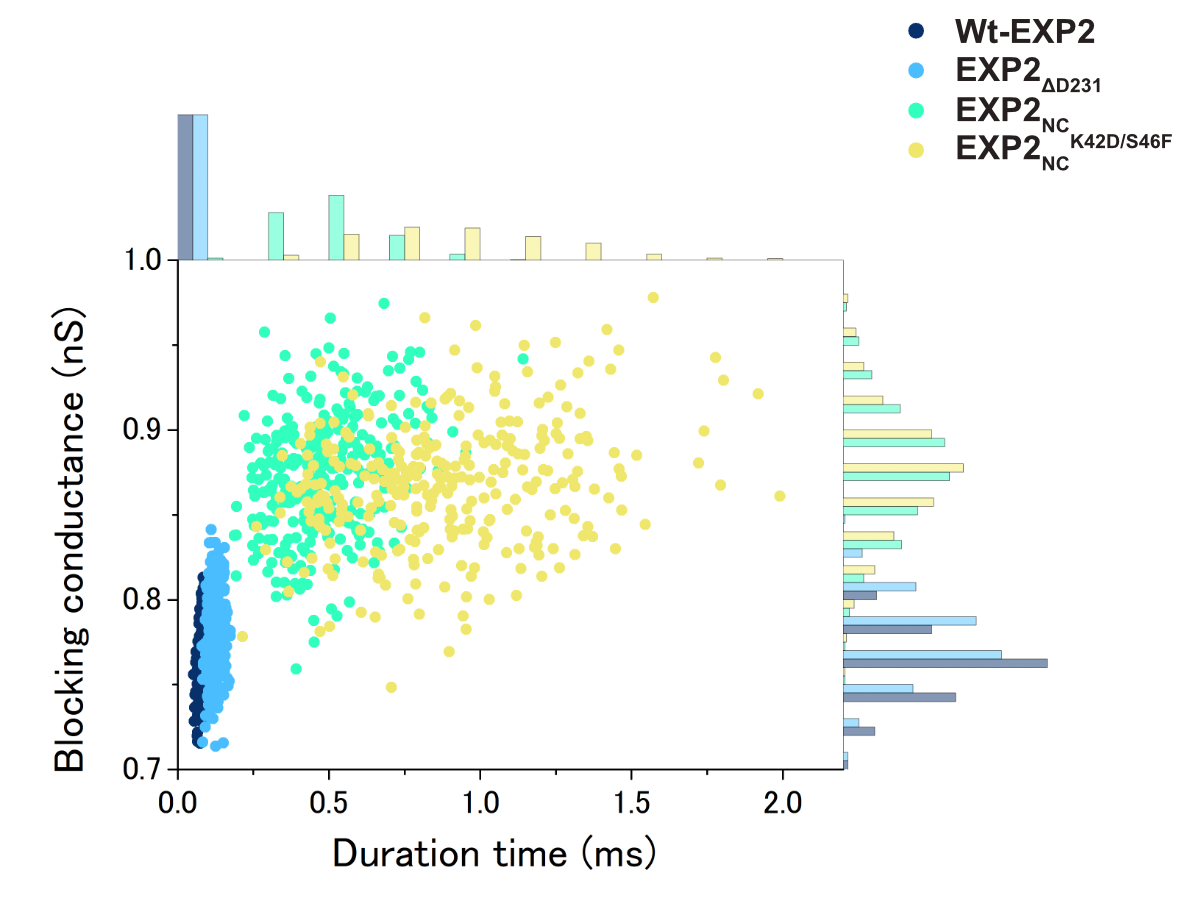
**

**Figure S4.** The scatter plot of the current noise properties of WT-EXP2 and its mutants.

**
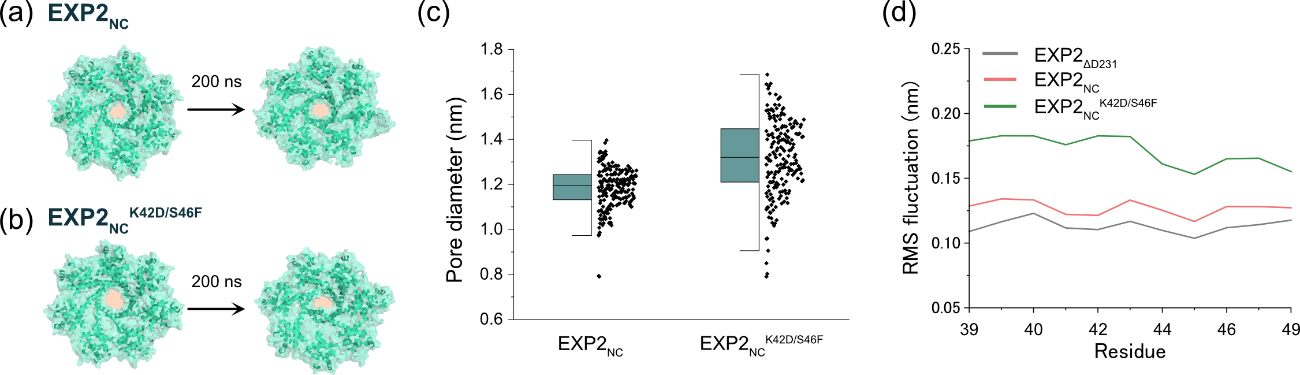
**

**Figure S5.** MD simulation of EXP2_NC_ and EXP2_NC_^K42D/S46F^. **(a-b)** The initial structure and the final structure of EXP2_NC_ **(a)**, and EXP2_NC_^K42D/S46F^ **(b)** for 200 ns MD simulation on the condition of DOPC membrane, 1 M KCl, at 395.15 K. **(c)** The pore diameter estimation in 200 ns MD simulation using HOLE software. **(d)** The analysis of RMSFCα of the transmembrane region (T39-49) of EXP2 mutants.

**
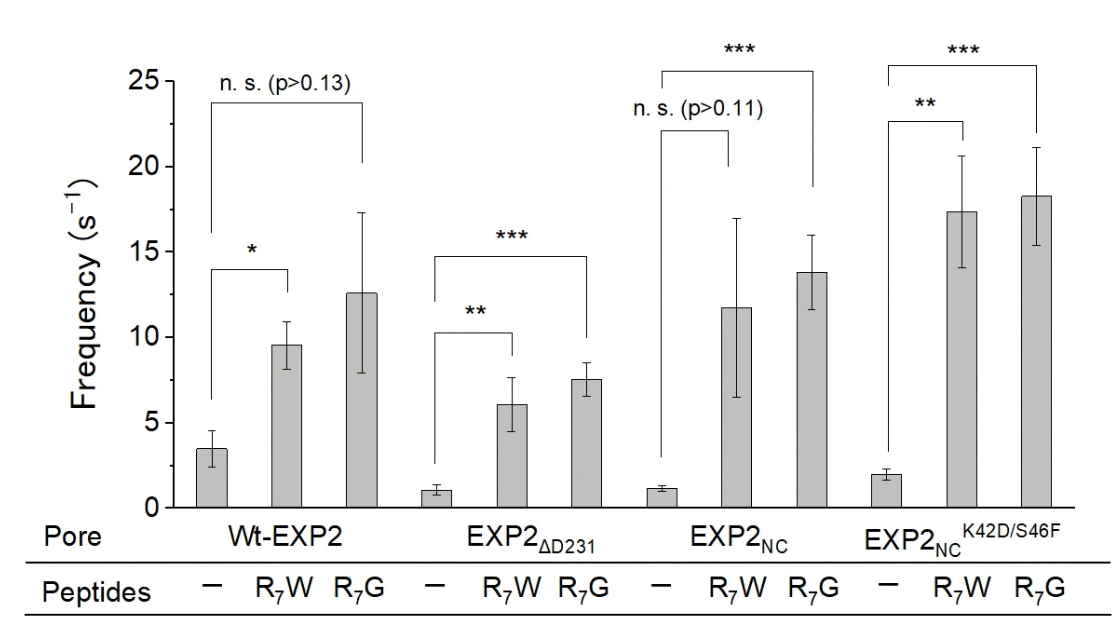
**

**Figure S6.** The event frequency of the blocking signals reflecting the detection of individual R_7_X peptides. Statistical significance is shown between current noise of each nanopore and each R_7_X peptide (*: 0.01<p<0.05; **: 0.001<p<0.01; ***: p<0.001, n. s.: not significant).

**
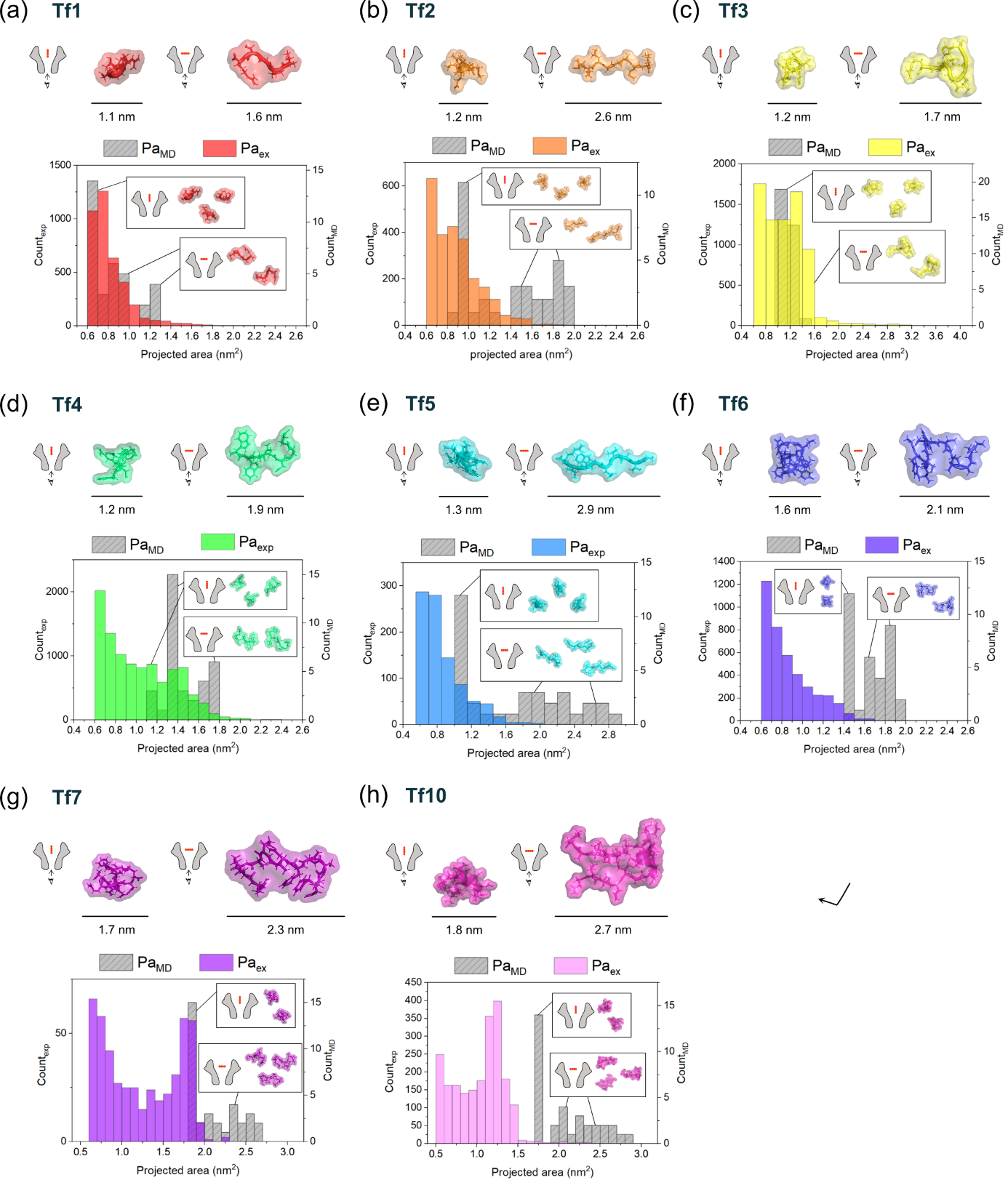
**

**Figure S7.** The projected area analysis of Tf peptides. **(a-h)** (Top) The shape when the peptide passes through the nanopore from the vertical direction (left), and the lateral direction (right). (Bottom) Comparison of the projected area histograms between the estimated from MD simulation result (dashed gray) and calculated from the experiment of each Tf peptides using EXP2_ΔD231_ nanopore. Data were recorded at 22 ± 2 °C and +100 mV in 1 M KCl, 10 mM citric acid, pH 3.8, using a 10 kHz low-pass Bessel filter with a 50 kHz sampling rate.

**Table S1.** Amino acid sequence of WT-EXP2 and its mutants. Light green shows the assembly strand domain of EXP2 nanopore.

|  | **Sequence** |
| --- | --- |
| **WT-EXP2** | VCDNGYGDAATSALTTVKDPISLTIKIYEHGVKNPTKIIHKLKKIRYRKVLRWRMWWVLLVRIVGDNTIEKTEKALREIWQCTIAVYNNLNAVESKPLFLHGILNECNNFATKLRQPSLIVAKIDIIKSQIYRFVSEPYLKIGSHTLYTHITDAVPQLPKETLKHLSSYMEKLKSMESKNIESGKYEFVDSSETDSTDDGKPDDDDDDDNFDDDDFDDDTVEEEASGDLFKNEKDENKE |
| **EXP2_ΔD231_** | VCDNGYGDAATSALTTVKDPISLTIKIYEHGVKNPTKIIHKLKKIRYRKVLRWRMWWVLLVRIVGDNTIEKTEKALREIWQCTIAVYNNLNAVESKPLFLHGILNECNNFATKLRQPSLIVAKIDIIKSQIYRFVSEPYLKIGSHTLYTHITDAVPQLPKETLKHLSSYMEKLKSMESKNIESGKYEF |
| **EXP2_NC_** | GDLAATSATTVIKDPISTIKDIYEHGKNPFTKIIHLKKFIRYRKLRWSRMWWVLVREIVGDNIEKKTEKALEIWDQCTIAYNNTLNAVEKPLLFLHGINECRNNFATLRQDPSLIVKIDQIIKSQYRFWVSEPYKIGRSHTLYHITPDAVPQPKECTLKHLSYM |
| **EXP2_NC_^K42D/S46F^** | GDLAATSATTVI**D**DPI**F**TIKDIYEHGKNPFTKIIHLKKFIRYRKLRWSRMWWVLVREIVGDNIEKKTEKALEIWDQCTIAYNNTLNAVEKPLLFLHGINECRNNFATLRQDPSLIVKIDQIIKSQYRFWVSEPYKIGRSHTLYHITPDAVPQPKECTLKHLSYM |

**Table S2.** Amino acid sequence of synthetic trypsinized fragments from lysozyme including their mass, and total charge at pH7.0 and pH 3.8. C_m_ means an alkylation of the cysteine residues.

| Molecule | Mass (Da) | Charge  (pH 7.0) | Charge  (pH 3.8) |
| --- | --- | --- | --- |
| Tf1 (TPGSR) | 517.3 | +1 | +1 |
| Tf2 (C_m_ELAAAMK) | 893.4 | 0 | +1 |
| Tf3 (HGLDNYR) | 874.4 | 0 | +2 |
| Tf4 (WWC_m_NDGR) | 993.4 | 0 | +1 |
| Tf5 (GTDVQAWIR) | 1046 | 0 | +0.7 |
| Tf6 (GYSLGNWVC_m_AAK) | 1325.6 | +1 | +1 |
| Tf7 (FESNFNTQATNR) | 1429 | 0 | +1 |
| Tf10(NLC_m_NIPC_m_SALLSSDITASVNC_m_AK) | 2508 | 0 | +1 |
